# Rainfall-Adaptive Landslide Monitoring Framework Integrating FLAC3D Numerical Simulation and Multi-Sensor Optimization: A Case Study in the Tianshan Mountains

**DOI:** 10.3390/s25175433

**Published:** 2025-09-02

**Authors:** Xiaomin Dai, Ziang Liu, Qihang Liu, Long Cheng

**Affiliations:** 1 School of Traffic and Transportation Engineering, Xinjiang University, Urumqi 830017, China; xmdai@xju.edu.cn (X.D.); chenglong@xju.edu.cn (L.C.); 2 Xinjiang Key Laboratory of Green Construction and Maintenance of Transportation Infrastructure and Intelligent Traffic Control, Urumqi 830017, China; 3 School of Mechanical Engineering, Xinjiang University, Urumqi 830046, China; 107552303314@stu.xju.edu.cn

**Keywords:** landslide monitoring, numerical simulation, FLAC3D, rainfall-induced landslides, sensor optimization

## Abstract

Traditional landslide monitoring systems struggle to capture the spatiotemporal dynamics of rainfall-induced hydro-mechanical processes, with a significant risk of signal loss during critical “unsaturated-saturated” state transitions. To address this issue, we propose an integrated framework that utilizes FLAC3D numerical simulation to dynamically optimize multi-sensor deployments. Through coupled seepage-stress analysis under different rainfall scenarios in China’s Tianshan Mountains, this study achieved the following objectives: (1) risk-based sensor deployment by precisely identifying shallow shear strain concentration zones (5–15 m) through FLAC3D simulation (with FBG density of 0.5 m/point in the core sliding belt and GNSS spacing ≤ 50 m); (2) establishment of a multi-parameter cooperative early warning system (displacement > 50 mm/h, pore water pressure > 0.4 MPa, strain > 6400 με), where red alerts are triggered when at least two parameters exceed thresholds, reducing false alarm rates; and (3) development of an adaptive sampling framework based on three rainfall intensity scenarios, which increases measurement frequency during heavy rainfall to capture transient critical points (GNSS sampling rate enhanced to 10 Hz). This approach significantly enhances the capture capability of critical hydro-mechanical transition processes while reducing the monitoring redundancy. The framework provides a scientifically robust and reliable solution for slope disaster-risk prevention and management.

## 1. Introduction

Landslides are a highly destructive type of geological disaster, particularly common in mountainous areas, where extreme weather events and periodic runoff intensify slope instability [1]. According to the Sixth Assessment Report of the Intergovernmental Panel on Climate Change, the frequency of extreme precipitation events in mid-latitude mountainous regions has increased by 35% since 2000, which is associated with a rise in landslide occurrences. In China alone, rainfall-triggered landslides caused more than 1200 deaths and economic losses exceeding 100 billion yuan between 2010 and 2022 [2]. Changes in precipitation patterns caused by climate change have led to a dramatic global increase in the frequency and scale of landslides, posing severe threats to infrastructure and human safety [3,4]. Because landslides arise from fragmented and weakened material cohesion, as well as mining activities and hydrogeological factors, traditional monitoring methods, such as manual inspections and fixed sensor networks, struggle to capture the spatiotemporal dynamics of the hydro-mechanical coupling processes induced by rainfall, particularly during rapid phase transitions between unsaturated and saturated states [5]. During heavy rainfall events, conventional sensors often miss critical pore water pressure signals owing to insufficient sampling frequency, leading to delays in early warning [6].

In recent years, breakthroughs in numerical simulation technology have provided new ways to address these difficulties. Three-dimensional finite-difference models offer a complete examination of stress–strain responses, displacement velocities, and pore water pressure dynamics, providing vital insights into the causes of landslide failure [7]. Basharat et al. [8] stress the importance of in-depth research and the employment of accurate methodologies for hazard and risk assessment, including numerical simulations and continuous monitoring of landslides. This highlights the existing gaps in current approaches and the necessity for further studies. Notably, Xu et al. [9] devised a discrete element model (DEM) parameter inversion method that improves the accuracy of landslide simulations by improving the contact parameters. Similarly, the free-fall cone penetration test of the soft seabed in the northern Baltic Sea showed that the dynamic combination of numerical simulation and field monitoring data can significantly improve the capture ability of key hydrodynamic mutation processes [10].

Current research primarily focuses on model validation or static susceptibility assessment; however, critical gaps persist in bridging numerical simulations with practical monitoring frameworks. Four core limitations require urgent attention:

(1) Dynamic Integration Deficiency: Simulation results are rarely utilized to dynamically guide sensor deployment or early warning thresholds [11]. This disconnect impedes the capture of transient hydromechanical transitions, particularly during unsaturated-saturated phase changes, where signal loss is frequent, consequently leading to delayed warnings [12]. (2) Static Sampling Methods: Conventional fixed-interval sampling methods cannot adapt to the acceleration of rainfall-induced deformation processes [13]. Field studies have shown that during intense rainfall, low-frequency GNSS measurements may miss critical precursor displacement rates exceeding 20 mm/h [14]. (3) Neglect of Spatial Optimization: The Spatial heterogeneity of rainfall infiltration and its control over local failure mechanisms are seldom incorporated into the design of sensor networks. Consequently, high-risk zones predicted by simulations (e.g., shear bands at depths of 5–15 m) lack targeted instrumentation, thereby reducing monitoring efficiency [15]. (4) Gap in Parameter Utilization: Key simulation outputs are not systematically translated into a basis for sensor type selection or placement density optimization. This underutilization diminishes the predictive capacity of monitoring systems [16].

In landslide monitoring, the precise positioning of sensors is crucial for acquiring key data related to surface displacement and the failure mechanisms. For instance, research on the failure mechanisms of mining landslides has underscored the importance of sensor layout, with results indicating that the placement method of earth pressure sensors significantly influences the accuracy of failure predictions [17]. Similarly, studies combining centrifuge model tests with finite element numerical simulations have shown that the strategic placement of sensors facilitates a deeper understanding of slope deformation behavior during landslides [18]. An experimental study employing the Finite Element Method (FEM) determined the optimal sensor configuration through a parametric analysis, which significantly enhanced the nondestructive inspection performance of embedded sensors [19]. This approach highlights the necessity of integrating numerical simulations into sensor layout design to achieve more reliable landslide detection and assessment. Furthermore, optimizing the sensor layout plays a pivotal role in identifying the most efficient and cost-effective sensor placement locations, thereby enhancing early warning systems for landslide hazards [20]. Advanced optimization techniques aim to improve the stability, accuracy, and efficiency of structural health monitoring and landslide prediction [21]. These methods facilitate the systematic determination of sensor placement schemes that maximize information acquisition while minimizing resource consumption. Such optimization is vital for enhancing sensor performance, which, in turn, indirectly promotes landslide monitoring capabilities by enabling more precise data collection [22,23].

However, predicting slope failure remains highly challenging for the following two key reasons: (1) Spatiotemporal Heterogeneity: The geotechnical and hydrological properties of slopes exhibit significant spatial variability (e.g., differences in permeability between soil and rock) and temporal nonlinearity (e.g., delayed rise in groundwater levels under continuous rainfall). These heterogeneities make uniform monitoring strategies difficult to apply, necessitating reliance on local high-resolution simulations to avoid false alarms [24]. (2) Unsaturated-Saturated Transition Risk: During rainfall infiltration, the transition from unsaturated to saturated states often leads to abrupt changes in the matric suction and pore water pressure. Conventional fixed-frequency sensors may miss these critical signals because of delayed response times [25].

To address this key challenge, this study proposes an integrated framework that innovatively utilizes the FLAC3D 7.0 numerical simulation for spatiotemporal and multiparameter monitoring. Specifically, the framework dynamically couples the seepage and stress fields to analyze complex mechanical responses and hydrological processes within slopes, thereby optimizing multi-sensor deployment strategies. Through simulation analyses under various rainfall scenarios in the Tianshan Mountains, China, this study achieved the following objectives: (1) identification of high-risk zones based on displacement, pore water pressure, and strain fields to enable precise spatial optimization of sensor layouts; (2) introduction of a rainfall-triggered adaptive sampling mechanism that increases the sampling frequency to 10 Hz during heavy rainfall to capture transient critical signals; and (3) establishment of a multiparameter early warning mechanism that integrates displacement, pore water pressure, and strain thresholds to enhance warning accuracy. The FLAC3D 7.0 framework not only resolves spatiotemporal heterogeneities within the rock and soil mass (such as variations in permeability and stress concentration zones) and tracks transient saturation fronts during the unsaturated-saturated transition, but also enables high-resolution monitoring of pore pressure, displacement and shear strain. This facilitates an optimized sensor configuration and effectively reduces the false alarm rate. This approach significantly enhances the capability to capture critical hydro-mechanical transition processes while reducing monitoring redundancy, offering a scientific, reliable, and forward-looking technical solution for slope disaster risk prevention and management [26].

## 2. Study Area

### 2.1. Geographical Location

**Figure 1:**
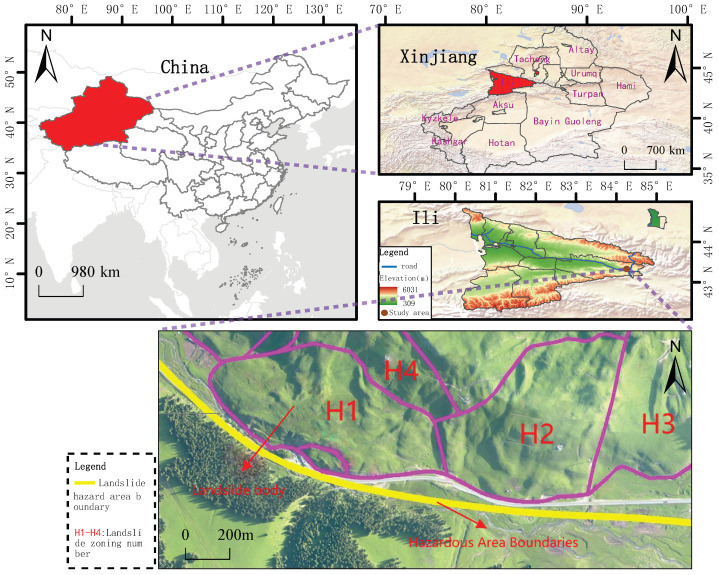
Schematic diagram of the location of the study area.

The research area is situated in the Xinjiang Uyghur Autonomous Region, covering parts of Xinyuan County in the Ili Kazakh Autonomous Prefecture and Hejing County in the Bayingolin Mongolian Autonomous Prefecture, deep in the Tianshan Mountains, extending west to east with complex topography, well-developed river systems, gully networks and incised terrain. Adverse geological conditions, such as landslides, pose significant risks to road safety and personnel. As depicted in Figure 1, the landslide is located on the right bank (downstream) of the Gongnaisi Valley along the G218 National Highway at coordinates 8446′17.3″ E, 4313′19.1″ N, featuring a banana-leaf-shaped planar morphology and a chair-like back scarp, divisible into four primary zones, with a rear escarpment height of approximately 30 m, a main axis length of approximately 1460 m, and a principal sliding direction of 233.

### 2.2. Meteorological Conditions

Owing to the impact of geography, moist air currents from the Arctic and Atlantic Oceans can reach this region through the western entrance of the Ili Valley. Upon reaching the Konis Valley, these air currents face the barrier formed by mountains on the north, east, and south sides, resulting in relatively plentiful precipitation and thus exhibiting the characteristics of a humid continental mid-temperature climate. The winter and summer seasons are longer, whereas spring and autumn are shorter, with plentiful sunshine and abundant light and heat supplies. Among the four seasons, spring is characterized by a rapid rise in temperature, summer is pleasant and rainy, autumn experiences a quicker drop in temperature, and winter is longer and colder.

The landslide area is characterized by a typical humid, continental, mesothermal climate. However, within the Gongnaisi Valley, affected by warm and moist airflows emanating from the Yili Valley, a small semi-humid continental mesothermal microclimate zone has emerged. This microclimate exhibits less marked seasonal changes, with spring and summer merging into a continuous growing season. The summer period is defined by relatively high precipitation, with a maximum annual average rainfall of 880 mm, while in some years, the total precipitation has reached a historical high of 1140.8 mm. The maximum daily rainfall recorded is 44 mm, with a typical daily rainfall intensity of 20 mm.

## 3. Formation Conditions and Influencing Factors of Landslides

Landslides are a consequence of the combined influence of endogenic and exogenic geological processes, involving a complex coupling of the mechanical properties of rock and soil masses, hydrological conditions, and topographical factors [27]. These factors include geography, geological structures, rock and soil composition, hydrometeorological conditions, human activities, and dynamic risk aspects [28]. The combination of these elements leads to the downward movement of soil or rock masses along weak or structural planes under the impact of gravity, ultimately culminating in landslides [29].

### 3.1. Geometric Morphology of Landslides

**Figure 2:**
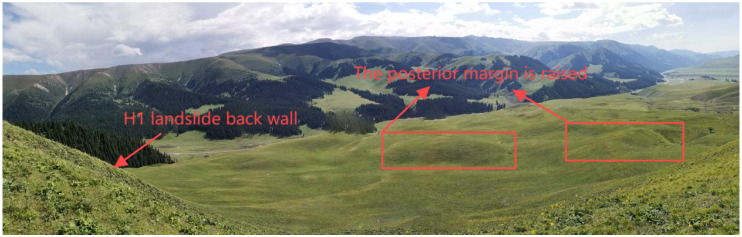
Landslide geometry.

The geometric morphology of landslides is characterized by their planar distribution and profile structure, collectively constituting their spatial identification markers [30]. The planar forms generally exhibit a distinctive “armchair” or tongue-like structure, where the arcuate boundary of the posterior margin contrasts dramatically with the tongue-shaped extension of the leading edge in the primary sliding direction [31]. Taking the Aken Daban H1 landslide as an example (Figure 2), its unique “plantain fan shape” planar distribution features a lateral width of up to 60 m and a longitudinal extension of approximately 70 m, with a main sliding azimuth of 30°, revealing the control of regional tectonic stress fields on the development direction of the landslide. The profile morphology is defined by a typical four-element structure: the steep inclination of the rear scarp typically surpasses 60°, and the thickness of the landslide body demonstrates significant spatial variation (the thickness of the H1 landslide varies from 8.1 to 14.9 m).

### 3.2. Structural Characteristics of Landslides

**Figure 3:**
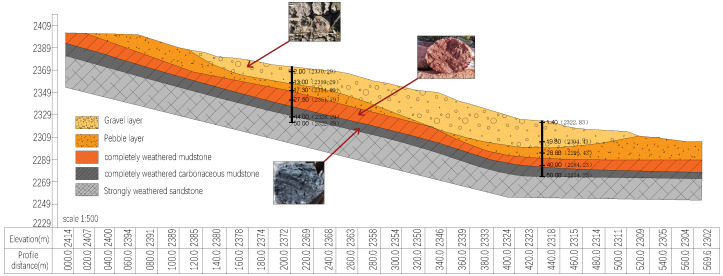
H1 landslide profile.

**Figure 4:**
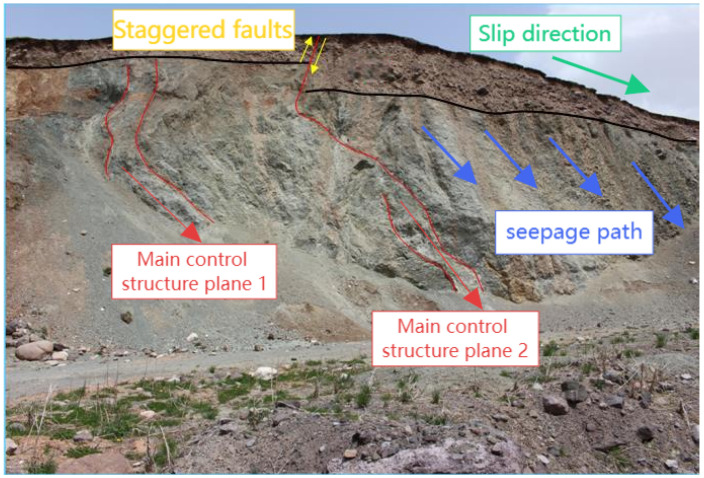
Block and gravel fault zones.

The three-dimensional spatial layout of landslide formations significantly influences deformation and evolution. As shown in Figure 3, the slip zone consists of completely weathered carbonaceous mudstone acting as a weak interlayer, underlain by stable, strongly weathered sandstone bedrock, which forms the slip bed and facilitates slip surface development. The landslide body, particularly in the H1 case, was composed of a heterogeneous mix of boulders, gravel, and weathered mudstone in its upper portion, with high spatial variability. With a permeability coefficient of approximately $10^{-4}\;m/d$, this material is highly susceptible to matric suction loss during rainfall. Combined with the fault zone influence, these conditions may trigger shallow landslides, as illustrated in Figure 4.

**Figure 5:**
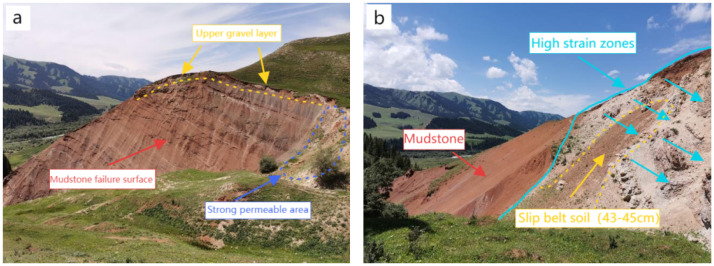
Structural characteristics of mudstone faults and overlying slippery soil layers: (**a**) Mudstone fault zone with sliding bed; (**b**) slippery soil layer.

The slip zone, as a critical weak layer, decisively affects the engineering geological behavior. In the H1 landslide, at depths of 43–45 cm, the cohesion dropped below 2 kPa, and the friction angle fell under 16°, forming a distinct strain-softening zone (Figure 5). The hydrological properties of the slip bed also play a key role in slope stability. The underlying bedrock exhibits stable geotechnical characteristics, with permeability on the order of $10^{-7}\;m/d$, cohesion up to 60 kPa, and internal friction angles exceeding 45°.

This heterogeneous structure promotes pore water pressure buildup at the soil–rock interface during rainwater infiltration, leading to hydrologically driven sliding along the interface and ultimately causing landslides.

### 3.3. Signs of Deformation on the Surface

**Figure 6:**
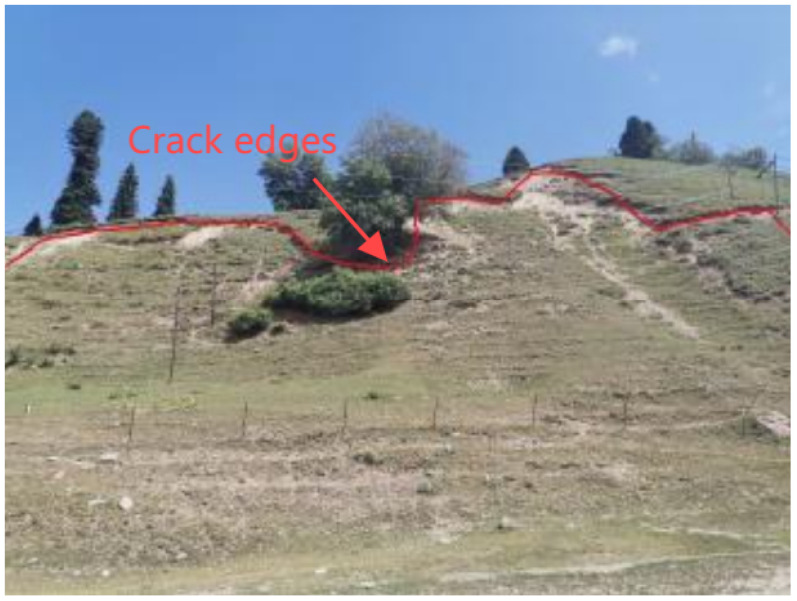
Tensile cracks in landslides.

The surface deformation field is a vital representation of landslide dynamics. The formation of tension crack networks at the back edge has substantial consequences for different evolutionary stages [32]. As shown in Figure 6, in the instance of H3 landslide, the fracture propagation rate near the 30-m-high scarp at the rear wall reaches 2 cm/day. These cracks extended at an intersection angle of 85°–95° relative to the major sliding direction, commensurate with the orientation of the maximum principal stress under plane strain conditions.

At the landslide toe, the formation of tongue-like structures due to compressional uplift not only alters the local topography, such as the 10-m uplift observed at the toe of the H3 landslide, but also reflects the spatial distribution of shear resistance along the sliding surface through accumulated volumetric strain.

Furthermore, the en echelon pattern of the lateral shear cracks reflected the dynamic evolution of the shear stress field along the landslide margins. Variations in the conjugate shear angles provide insights into the adjustment of the stress state during the sliding process, offering crucial limits on the mechanical behavior and kinematic evolution of the landslide [33].

### 3.4. Hydrogeological Characteristics

Hydrological and geological interactions are key dynamic components that influence landslide stability. Variability in surface water infiltration produces dynamic oscillations in the saturation field within the landslide mass [34]. During the rainy season, the volumetric water content of the H1 landslide can reach up to 32%, approximately 85% of its saturated water content, which greatly reduces the matric suction. Variations in groundwater levels demonstrate a time-lag effect in decreasing the mechanical characteristics of the sliding zone. Field data from the H3 landslide suggest that a 2-m elevation in the groundwater level may contribute to a reduction in effective stress within the sliding zone by up to 40 kPa. Furthermore, turbidity changes at seepage outlets near the slope toe are closely associated with fine-particle migration within the sliding zone, highlighting their usefulness as an early warning indicator for sliding surface activation.

### 3.5. Comprehensive Mechanism and Main Influencing Factors of Landslide

The integrated mechanism of landslides is derived from the coupled effects of various elements, including geological structural fragility, hydro-meteorological conditions, topographic characteristics, and human activities. The primary mechanism is the loss of geotechnical qualities generated by rainfall infiltration [35]. This process is principally manifested by a significant reduction in both cohesion (c) and internal friction angle (ϕ), accompanied by a sharp increase in pore water pressure, which collectively diminishes the shear resistance of the slope.

The key factors impacting the results include:

1. Geological factors: These include the types of lithology and soil (slick mudstone and highly weathered rock masses), the development of structural planes (such as faults and joints), and the spatial distribution of weak interlayers, all of which significantly influence the mechanical behavior and failure mode of slopes [36];
2. Topographic considerations: Steep slopes ranging from 30° to 60°, combined with elevation differences of 10 to 200 m, exacerbate gravity erosion and stress concentration, hence increasing the susceptibility to slope instability [37];
3. Hydro-meteorological factors: Strong rainfall leads to soil saturation, causing transient seepage zones that can trigger either progressive or rapid slope failure by reducing effective stress and raising pore water pressure [38];
4. Geotechnical parameters: The dynamic deterioration of shear strength, notably the time-dependent drop in cohesion (c) and internal friction angle (ϕ), plays a vital role in slope stability. In addition, fluctuations in soil unit weight due to moisture content changes directly alter the driving forces responsible for slope movement [39].

**Figure 7:**
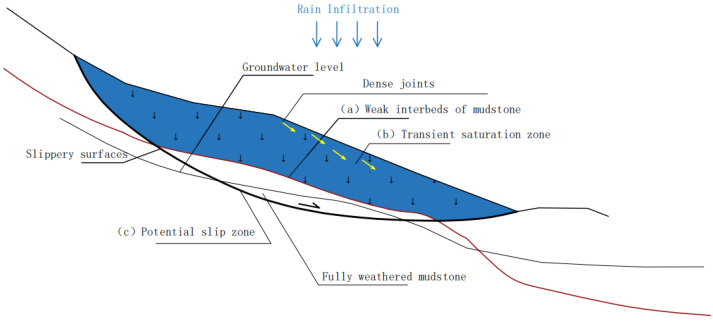
Schematic diagram of the slip mechanism.

As shown in Figure 7, the major regulating interface of the landslide is the contact zone between the weak carbonaceous mudstone interlayer (highlighted by the red dashed line in the image) and the overlying severely weathered rock mass. This sliding surface (a) exhibited a terraced morphology owing to faulting, which resulted in a considerable drop in shear strength along this plane.

The joint-rich zones indicated by the yellow arrows further improve rock mass fragmentation, providing favorable channels for rainfall infiltration. Seasonal precipitation infiltrates through surface fissures and collects at the top of the mudstone layer, generating a transitory saturated zone (b), as represented by a blue-shaded area.

The subsequent rise in pore water pressure reduces the effective stress, prompting flow-like failure within the soil mass located in the possible slip zone (c), as highlighted by the white dashed line in the figure.

### 3.6. Analyze the Implications

The design of a landslide monitoring plan must be based on a thorough understanding of the geological properties and dynamic evolution mechanisms of the slope to ensure scientific validity and effectiveness. Taking the H1 landslide as an example, its layered structural features and poor mechanical properties of the slip zone—characterized by cohesion c≤20 kPa and internal friction angle $\phi \leq 16^{\circ }$—indicate that the slope is in a condition of critical stability.

To solve this problem, a three-dimensional elastoplastic model can be built using FLAC3D 7.0. By including the mechanical properties of the slip zone, the model enables a precise prediction of pore water pressure dynamics under changing rainfall intensities and their impact on slope stability. For instance, when the rainfall intensity surpasses 30 mm/day, the model displays a rapid increase in pore water pressure, which might directly drive slope instability.

By integrating geological features, mechanical parameters, and dynamic evolution processes, the monitoring technique should transcend phenomenon-based observation to mechanism-driven early warning, thereby establishing a sound scientific foundation for landslide hazard prevention and mitigation.

## 4. Numerical Simulation of Landslide Dynamic Response Analysis

In this study, FLAC3D 7.0 was used to simulate the mechanical reaction of geotechnical materials and their associated hydro-mechanical behavior. The main premise involves linking the seepage field with the stress field to achieve a dynamic simulation of landslide deformation processes. The H1 cross-section was selected to describe the landslide deformation mechanisms under various loading scenarios.

### 4.1. Multiphysics Coupling Mechanism and Model Building

#### Principles of Multiphysics Coupling

FLAC3D 7.0 employs an explicit time-marching finite-difference approach, discretizing the continuum into a set of grid cells. The equations of motion for each cell were solved incrementally based on Newton’s second law. The use of a Lagrangian formulation allows the grid cells to deform and move with the material, enabling the precise capture of massive deformations and material nonlinearity, such as those occurring during landslide instability processes.

The seepage analysis was based on Darcy’s law and the mass conservation equation, which solved the governing equation of the pore water pressure field as follows:

Seepage Field Simulation Principle [40]:

$$\frac{\partial }{\partial x_{i}}\left(k_{ij}\frac{\partial h}{\partial x_{j}}\right)+Q=S_{s}\frac{\partial h}{\partial t}$$

where $k_{ij}$ is the permeability tensor, *h* is the hydraulic head, *Q* is the source sink term, and $S_{s}$ is the specific storage coefficient. FLAC3D 7.0 mimics the unsaturated-saturated seepage process through dynamic coupling of nodal pressures and element permeability, reflecting the impacts of rainfall infiltration and pore water pressure fluctuations on slope stability.

Stress Field Simulation Principle [41].

The stress field analysis is based on the Mohr–Coulomb yield criterion and effective stress principle, and calculates the equilibrium equations for nodal displacements and element stresses as follows:

$$\sigma_{ij}^{\prime }=\sigma_{ij}+\alpha p\delta_{ij}$$

where $\sigma_{ij}^{\prime }$ is the effective stress, *p* is the pore water pressure, and α is the Biot coefficient (effective stress coefficient). The software iteratively calculates the stress–strain relationship of elements and dynamically records the development of plastic zones and displacement evolution, making it suited for simulating progressive slope failure.

### 4.2. Model Building

**Figure 8:**
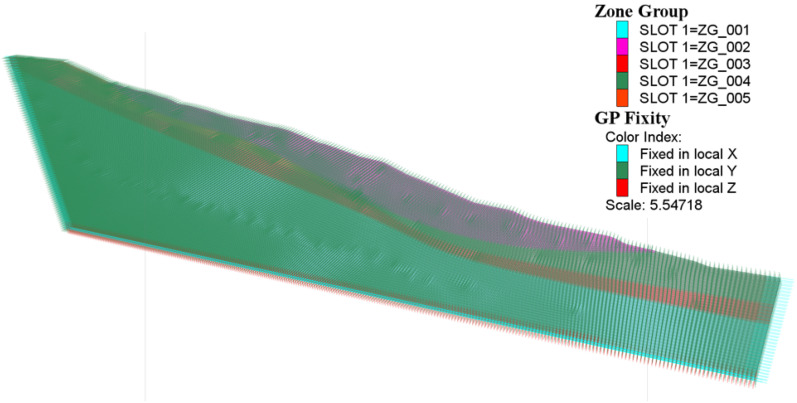
Model boundaries.

As shown in Figure 8, based on geological cross-sections and drill data from the research area, a three-dimensional slope geometry model was created to accurately define the stratigraphic boundaries, weak interlayers, and other realistic subsurface conditions. In this study, the 3D modeling framework for the landslide-prone central zone was first constructed using Rhinoceros 7 software and subsequently imported into FLAC^3D^ for the numerical simulation.

A consistent mesh with a grid spacing of 0.5 m was produced throughout the model. Using the zone group command, the model was separated into five discrete layers, with the boundary between the gravel and completely weathered mudstone layers explicitly designated as the weak shear zone.

Hydraulic and mechanical parameters were allocated to each stratigraphic unit according to field and laboratory data. Boundary conditions were specified as follows: full fixity (constrained in *x*, *y*, and *z* directions) at the bottom boundary; fixed *y*-direction movement on the left and right sides; and fixed *x*, *y*, and *z* movements on the front and rear borders to replicate laterally restricted circumstances.

#### Material Parameters

The internal parameter assignments in the model are based on experimental results obtained from field studies and laboratory tests, which encompass both strength qualities and structural parameters. Table 1 summarizes the important mechanical characteristics of the geotechnical materials used in the model, including the unit weight, elastic modulus, Poisson’s ratio, cohesion, and internal friction angle.

The hydraulic parameters of the model were developed based on data from the Hydrogeological Handbook of Xinjiang and laboratory permeability tests. As shown in Table 2, the key input factors included porosity, saturated volumetric water content, and saturated hydraulic conductivity.

Based on a detailed statistical analysis of historical meteorological data, the study region is characterized by a maximum recorded daily rainfall of 44 mm/day, with an annual average precipitation of approximately 20 mm/day. Notably, significant precipitation episodes have been recorded for up to 72 consecutive hours. These crucial hydrological metrics underline the region’s vulnerability to protracted rainfall episodes, which exert a considerable influence on slope stability and landslide initiation mechanisms.

In addition to modeling real field conditions, two unique extreme rainfall scenarios were constructed to examine the impacts of varying rainfall intensities and durations on slope hydrology and stability. These scenarios were created to reflect high-intensity short-duration and moderate-intensity long-duration rainfall events, both of which are known to be significant causes of slope failure under different antecedent moisture levels.

Scenario 1—High-Intensity Rainfall: A continuous rainfall event with an intensity of 44 mm/day (matching the maximum observed daily rainfall) was applied for a duration of 72 h. This scenario models strong storm conditions that can lead to rapid infiltration, considerable pore water pressure buildup, and a corresponding loss in the shear strength of the slope materials.

Scenario 2—Moderate-Intensity Rainfall: A persistent rainfall of 20 mm/day (equal to the annual average daily rainfall) was applied continuously for 72 h. This scenario depicts more common yet persistent soaking conditions, which may gradually saturate the subsurface and slowly impair slope stability over time.

Together with the baseline conditions, the developed rainfall scenarios provide a strong framework for examining the hydro-mechanical response of slopes under varied climatic loading situations.

### 4.3. Dynamic Evolution of Slip Depth

**Figure 9:**
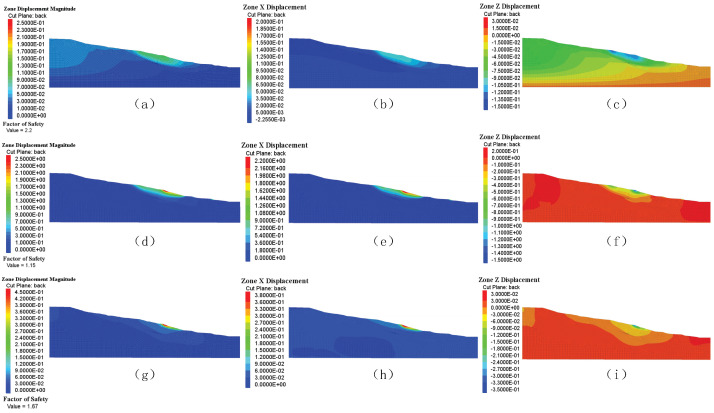
Displacement analysis: (**a**) total displacement of the natural state; (**b**) horizontal displacement of the natural state; (**c**) vertical displacement of the natural state; (**d**) total displacement of scenario 1; (**e**) horizontal displacement of scenario 1; (**f**) vertical displacement of scenario 1; (**g**) total displacement of scenario 2; (**h**) horizontal displacement of scenario 2; (**i**) vertical displacement of scenario 2.

As shown in Figure 9, the overall displacement magnitude of the slope under natural conditions exhibited a non-uniform distribution, with peak values concentrated near the surface. The highest total displacement at the crest was 14.2 mm, and it steadily diminished with increasing depth, approaching zero beyond 25 m. This pattern suggests the presence of a shallow deformation zone, predominantly dominated by surficial plastic flow. The safety factor determined from the analysis was 2.2, which, according to conventional geotechnical engineering criteria (safety factor > 1.5), indicates that the slope remains in a minimally stable condition.

From the standpoint of directional components, the horizontal (X-direction Figure 9b) displacement follows a parabolic distribution, with a maximum value of 5.37 mm occurring at depths between 10 m and 15 m, showing lateral spreading driven by gravity loading. In contrast, the vertical (z-direction; Figure 9c) displacement demonstrated a noticeable downward tendency, with the greatest settlement of 14.2 mm observed near the toe of the slope within the depth range of 5–10 m, consistent with compression-induced subsidence within the crucial sliding zone. Notably, the z-displacement switches direction (i.e., becomes negative) below 30 m, suggesting a probable upward heave induced by pore pressure accumulation or stress redistribution. These data collectively demonstrate the anisotropic deformation behavior of the slope, where horizontal spreading and vertical sinking interact to determine its stability.

As indicated in Figure 9d, under the greatest rainfall (Scenario 1), the safety factor of the slope is 1.15, nearing the critical threshold (safety factor ≥ 1.05), suggesting a potential risk of instability. Displacement field analysis revealed that the greatest surface displacement reached 2.442 m, demonstrating an exponential decrease with depth and approaching zero near the toe of the slope. The displacement magnitude may be categorized as follows: in the shallow layer (0–10 m depth), displacements surpass 1.0 m (with a peak of 2.442 m); between 10 and 30 m depth, displacements decrease to 0.2–1.0 m; and at depths more than 30 m, displacements fall below 0.2 m. These characteristics indicate that the landslide mechanism is driven by shallow, traction-type sliding, principally induced by the considerable weakening of surficial geotechnical materials owing to severe rainfall.

The horizontal (X-direction Figure 9e) displacement reached a maximum of 2.164 m at the slope crest, oriented outward from the slope, with an influence extending down to approximately 30 m depth. The model suggests that throughout the depth range of 0–20 m, the average displacement is 1.26 m; it drops progressively to 0.2–0 m between 20 and 30 m and becomes negligible below 30 m.

Vertical (Z-direction Figure 9f) displacement demonstrates notable subsidence features: surface settlement reaches 1.41 m and reduces with increasing depth, reducing to 0.2 m at 15 m depth. Notably, significant sinking occurs between the 0 and 10 m depth zone (displacement range: 0.2–1.5 m), whereas the subsidence becomes more gradual between 10 and 15 m (0.1–0.5 m). This sudden subsidence pattern reflects the likely presence of weak interlayers or localized collapse triggered by hydraulic infiltration within the slope body. Such vertical deformation synergistically interacts with horizontal displacement, further increasing the risk of slope instability.

As illustrated in Figure 9g, under the second average rainfall situation (Scenario 2), the displacement characteristics of the slope are predominantly defined by significant deformation in the superficial soil layers, whereas the deeper strata exhibit comparatively moderate deformation. The overall displacement, along with its X- (Figure 9h) and Y-directional (Figure 9i) components, was largely centered within the top 10–15 m depth. The greatest total displacement reached approximately 0.44455 m, with peak values of 0.37108 m in the X-direction and −0.0094687 m in the Y-direction (assuming downward as positive). The computed safety factor was 1.67, suggesting that the slope remained in a generally stable state according to standard geotechnical engineering criteria (safety factor > 1.5). However, significant attention should still be focused on the deformation behavior of superficial soil layers to ensure long-term slope stability. Notably, the substantial X-direction displacement was similarly confined to the near-surface zone to approximately 10–15 m in depth.

### 4.4. The Response Law of Seepage Field

#### 4.4.1. Pore Water Pressure Change Response

**Figure 10:**
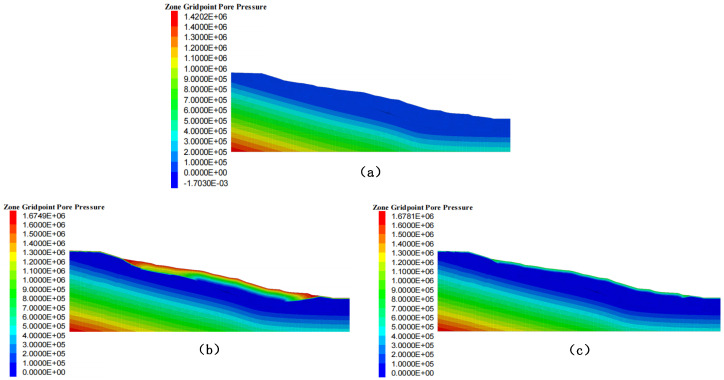
Pore water changes: (**a**) Initial pore water, (**b**) Pore water change in Scenario 1, (**c**) Pore water change in Scenario 2.

The FLAC3D 7.0 simulation results (Figure 10) revealed the spatiotemporal evolution patterns of the pore water pressure in the slope under different rainfall intensities.

In the baseline natural state (Figure 10a), the pore water pressure exhibits a hydrostatic pressure distribution. The shallow layer (0–5 m) showed negative pressure (matric suction), whereas the pressure in the deep layer (>20 m) approached zero, with a maximum pressure gradient not exceeding 0.05 MPa/m.

In the high-intensity rainfall scenario (Scenario 1) (Figure 10b), a dramatic change occurred in the shallow layer. Within 6 h of rainfall, the pore water pressure in the shallow layer (0–10 m) rapidly increased from its initial negative value to 0.1–0.5 MPa, forming a significant positive pressure zone. The core area of high pressure (>0.4 MPa) was concentrated at a depth of 5–8 m, with a horizontal extent of approximately 30 m (in the middle-front part of the landslide). The pressure attenuates to below 0.1 MPa at a depth of 15 m and shows no significant change below 20 m, indicating that the depth of infiltration influence is limited to the shallow layer (<20 m).

Under continuous moderate-intensity rainfall (Scenario 2) (Figure 10c), a significant cumulative effect was observed. The pressure increase in the shallow layer was minimal (<0.1 MPa), but persistent pressure accumulation occurred in the deep layer (20–50 m). The pressure contours displayed a nearly horizontal, layered distribution, reflecting a uniform infiltration process, with an influence depth extending to more than 40 m.

#### 4.4.2. Response to Soil Saturation Change

**Figure 11:**
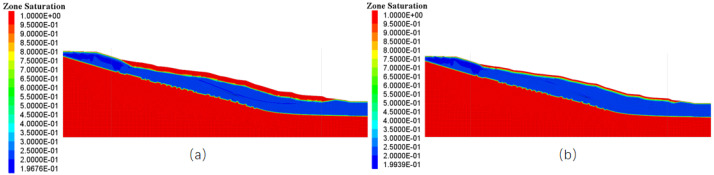
Soil saturation change: (**a**) the change of soil saturation under working Scenario 1, (**b**) the change of soil saturation under working Scenario 2.

In Figure 11, FLAC3D 7.0 simulations demonstrate that a severe rainfall event of 44 mm (Scenario 1, Figure 11a) generated significant shallow saturation in the slope. The surface layer (0–2 m depth) approached complete saturation (saturation degree $S_{r}=1.00$), with a saturated zone ($S_{r}\geq 0.95$) continuing below to approximately 6 m depth, where the saturation gradually declined from 1.00 to 0.95. Below this saturated front, a high-moisture transition zone persists, maintaining saturation levels between 0.85 and 0.95 at depths of 6–15 m (e.g., ∼0.85 at 10 m). These data show that severe rainfall causes localized transitory wedges of pore water near the surface of the soil. However, deeper soils remain unsaturated; at a depth of 30 m, the saturation is only 0.45, demonstrating that infiltration under heavy rainfall is predominantly vertical and restricted by short-term infiltration capacity. The maximum influence depth is approximately 15 m, where saturation reaches 0.75–25% higher than in deeper strata.

In contrast, moderate rainfall of 20 mm (Scenario 2, Figure 11b) does not induce full surface saturation (surface saturation remains at $S_{r}=1.00$), but encourages more homogeneous moisture migration into deeper layers. While the saturated zone ($S_{r}\geq 0.95$) extends to a comparable depth (∼6 m) as in Scenario 1, noteworthy differences arise in the mid- to deep zones. At depths of 15–30 m, the saturation values in Scenario 2 are systematically higher than those in Case One (for example, 0.60 vs. 0.55 at 20 m). Particularly at the crucial depth of 35 m, saturation reaches 0.40 in Case Two, showing a 14.3% increase compared to 0.35 in Case One. This suggests that low-intensity rainfall slowly lifts the wetting front via persistent infiltration. This “deep progressive saturation” extended down to 40 m depth (with $S_{r}=0.25$), indicating the power of low-intensity rainfall to drastically modify the deep seepage field. Although it lacks the surface saturation effects of strong rainfall, long-term infiltration increases the effective wetting depth by approximately 67% (relative to the 24 m wetting depth in Scenario 1).

### 4.5. The Response Law of the Stress Field

**Figure 12:**
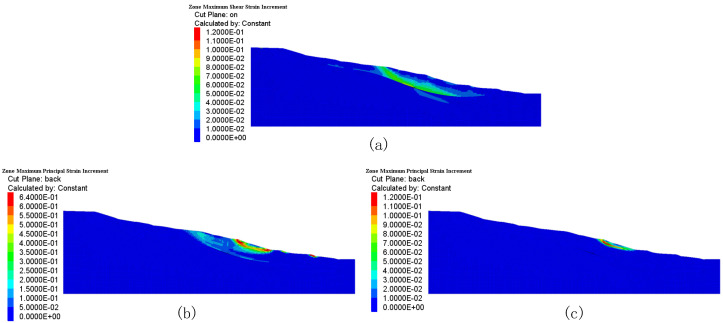
Stress field changes: (**a**) natural state stress field, (**b**) Scenario 1 stress field change, (**c**) Scenario 2 stress field change.

In Figure 12, the numerical simulations demonstrate that rainfall considerably modifies both the amplitude and spatial distribution of the shear strain within the slopes. In Figure 12a, under natural conditions, the largest incremental shear strain is centered at the soil–rock interface between 20 and 30 m depth, with a peak value of $1.5\times 10^{-2}$ Pa. This pattern indicates gravity-driven deep-seated shear deformation.

In contrast, under the severe rainfall scenario of 44 mm (Scenario 1, Figure 12b), the shear strain concentration migrates toward shallower depths. A considerable shear strain arose in the surface layer (0–5 m), reaching a peak of $6.4\times 10^{-1}$ Pa, which is more than an order of magnitude larger than that under normal conditions. A high-strain zone (>30% relative increase) stretched down to a depth of 8 m. These results demonstrate that severe rainfall produces hydraulic softening of shallow soils, resulting in a shift of the shear zone from deep structural planes to the slope surface.

Under a moderate rainfall scenario of 20 mm (Scenario 2, Figure 12c), an intermediate strain pattern was observed: the peak shear strain ($8.7\times 10^{-2}$ Pa) occurred at a depth of 5 m, while strain increments ranging from $2.5\times 10^{-2}$ to $4.0\times 10^{-2}$ Pa persisted between depths of 5–10 m, which was 60–100% higher than those under natural conditions at the same locations. This indicates that infiltration triggers several shear planes across distinct stratigraphic layers via a linked seepage mechanism.

### 4.6. Data Validation Based on Sensitivity Analysis

Based on the numerical simulation results of FLAC3D 7.0, the sensitivity analysis of key parameters, such as pore water pressure and displacement, was further analyzed, and the influence of each input parameter on the dynamic response of the landslide was quantified, which provides a theoretical basis for the design of the monitoring system. In this section, local sensitivity analysis is used to evaluate the stability of the FLAC3D 7.0 simulation results by quantifying the simulation output changes caused by the disturbance of key geotechnical parameters (±20%) under the 44 mm/day rainfall scenario (Scenario 1).

#### 4.6.1. Sensitivity Analysis Framework

The model adopts the same geometry and boundary conditions as described in Section 4.2, but sets the key parameters as the variables. The key parameters were selected based on their influence on the hydraulic behavior of the landslide (Table 3).

The formula for the rate of change of the output indicator is as follows:

$$\delta =\frac{V_{p}-V_{b}}{V_{b}}\times 100\%$$

where δ is the relative rate of change of the output variable; $V_{p}$ is the output value after parameter perturbation;$V_{b}$ is the original output value under the baseline parameter.

#### 4.6.2. Sensitivity Analysis Results

**Figure 13:**
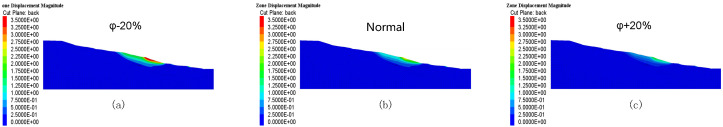
Internal friction angle sensitivity analysis diagram: (**a**) ϕ-20% slip response; (**b**) slip under normal conditions; (**c**) ϕ 20% slip response.

Taking the perturbation of the friction angle within the sliding zone as an example, its effect on the sensitivity of the displacement is shown in Figure 13.

As shown in the figure above, Table 4 shows the effects of the remaining parameter perturbations on the key output indicators.

The results indicate that the internal friction angle and rainfall intensity are the most sensitive parameters influencing landslide stability, which is consistent with the field monitoring and numerical findings reported by Liu et al. [42] on the dynamic response of twin parallel tunnels in unsaturated soil under metro train loading conditions. Zhang et al. [43] also confirmed that the internal friction angle is a key parameter controlling landslide stability.

When the internal friction angle decreases from $25^{\circ }$ to $20^{\circ }$ (a 20% reduction), the displacement increases by 33.0%. However, when the internal friction angle was further reduced to $20^{\circ }$, the displacement decreased by 26.0%, indicating that the model captured a critical threshold behavior associated with the internal friction angle. When the rainfall intensity reaches 52.8 mm/day, the saturation depth exceeds 18 m, approaching the depth of the deep-seated slip surface (20–30 m). This behavior aligns with the observed landslide responses under conditions of a “maximum daily rainfall of 44 mm” and “72-h continuous rainfall,” demonstrating that the model possesses significant predictive capability.

Based on real physical systems, the responses of different output indicators to the same parameter perturbation should be consistent. The following response chains were observed:

- 20% decrease in hydraulic conductivity → 20.0% increase in pore water pressure → increase in saturation depth
- 20% decrease in internal friction angle → 25.0% increase in displacement → increase in peak shear strain
- 20% increase in rainfall intensity → 23.3% increase in saturation depth → increase in displacement

These response chains are consistent with the fundamental principles of hydrology and soil mechanics, fully aligning with the established landslide mechanism: rainfall → infiltration → pore pressure rise → strength reduction → displacement increase. When multiple output indicators exhibit logically coherent and physically consistent responses to parameter perturbations, the model results can be deemed reliable.

## 5. Optimized Sensor Placement Based on Simulation Results

### 5.1. Monitoring Equipment Layout and Selection

#### 5.1.1. General Guidelines for Sensor Deployment

Based on the dynamic response characteristics of landslides revealed by the FLAC3D 7.0 numerical simulation, general guidelines for sensor deployment were established in accordance with international geotechnical engineering monitoring standards (ISO 18674 series, ASTM D6429) [44,45]. As shown in Table 5, these guidelines cover three types of monitoring items: displacement, pore water pressure, and stress–strain.

#### 5.1.2. Sensor Type Matching Design

For the high-shear-depth zones identified by FLAC3D 7.0 simulations—typically corresponding to slip surfaces at depths of 5 m to 15 m—a synergistic monitoring strategy combining surface InSAR/GNSS and deep-buried fiber Bragg grating (FBG) inclinometers is generally implemented [46,47].

Surface deformation monitoring is performed using Interferometric Synthetic Aperture Radar (InSAR) technology, which provides millimeter-level accuracy (±2 mm), enabling the detection of small precursory deformations prior to slope failure. Meanwhile, GNSS stations operate in dual-frequency real-time kinematic (RTK) mode and are strategically deployed in both the tension-cracked zone at the landslide rear and the bulging zone at the toe, allowing for real-time updates of three-dimensional coordinates at key nodes with a sampling frequency.

Subsurface deformation was monitored using dispersed fiber Bragg grating (FBG) inclinometers deployed along a zigzag route aligned with the simulated main sliding direction. By applying wavelength shift demodulation techniques, these sensors provide continuous strain profiles along the depth axis with a spatial resolution of 0.5 m.

Vibrating-wire pore pressure transducers are deployed within zones of elevated sensitivity to excess pore water pressure—specifically, in regions near the saturated–unsaturated interface where the hydraulic gradient exceeds 0.5 kPa m^−1^. These transducers are installed at crucial nodes along the essential seepage pathways. Their long-term stability enables good monitoring of the dissipation process of excess pore pressure generated by rainwater infiltration.

To address the shear strain concentration zones observed in the FLAC3D 7.0 simulations, defined as locations where the greatest incremental shear strain exceeds 5%, a sensor array including fiber Bragg grating (FBG) strain sensors was deployed. The FBG sensors were linearly positioned along the potential sliding direction and monitored the accumulated plastic strain by wavelength shift inversion. Their fatigue-resistant qualities make them ideal for long-term static strain monitoring. By measuring the continuous accumulation of static strain, FBG sensors provide a full evaluation of both short-term triggering mechanisms and long-term deformation history [46].

#### 5.1.3. Sensor Adaptation Rules Based on Analog Data

The specific sensor deployment principles, informed by the characteristics of numerical simulations, adhere to the core logic of “risk-driven density, magnitude-defined accuracy, and slope-corrected positioning.” The detailed specifications are as follows (Table 6):

### 5.2. Space-Optimized Layout Strategy

#### 5.2.1. Displacement and Stress Field Monitoring Scheme

The specific sensor deployment principles, informed by the characteristics of numerical simulations, adhere to the core logic of “risk-driven density, magnitude-defined accuracy, and slope-corrected positioning.” The detailed specifications are as follows (Table 6): The surface displacement monitoring network should fully cover the entire landslide area, including the tension-cracked zone at the rear edge, sliding zone in the central-toe region, and bulging zone at the slope toe. Particular attention should be paid to dense sensor deployment within the surface projection of the crucial possible slip zone established through numerical simulations.

1. GNSS Monitoring System:
  The GNSS reference station should be installed in a stable area outside the landslide influence zone. Monitoring stations should be geographically classified according to geomorphic units as follows:
  - At least two monitoring stations should be positioned in the tension-cracked zone at the rear edge to quantify extensional deformation.
  - Three to five stations are required in the central-toe sliding zone to record the displacement vectors.
  - One to two stations should be established in the bulging zone to monitor uplift deformation.
  In the sliding zone, the spacing between neighboring GNSS stations should be maintained within 100 m, whereas in the rear edge and toe regions, the spacing should not exceed 500 m.
2. InSAR Data Acquisition:
  The InSAR coverage must entirely include the landslide-prone area to ensure complete visibility without occlusions or shadowing effects. Priority should be given to places where surface deformation is extremely sensitive along the radar line-of-sight (LOS) direction, thereby enhancing detection accuracy and temporal coherence.
3. Deep Fiber Bragg Grating (FBG) Stress Inclinometer Deployment:
  The deep FBG inclinometers should be vertically mounted in the primary sliding path predicted by computer calculations, extending downward through the probable slip zone at depths of 5–15 m. A zigzag trajectory with lateral offsets of 1–2 m should be employed to achieve full-thickness coverage of the shear zone. As shown in Table 7, the arrangement of its quantity is regulated
  Sensor points should be preferentially situated within the shear strain concentration zones found by FLAC3D 7.0 simulations—particularly within the core region of the major slip surface. Additional auxiliary sensors should be positioned near the interface between the sliding zone and the underlying bedrock to monitor interfacial deformation.
  The axial spatial resolution of the sensing system should exceed 0.5 m, with one FBG sensor inserted every 0.5 m along the borehole. Within the main slip zone, the horizontal separation between consecutive boreholes should not exceed 5 m, but in peripheral areas, this spacing can be extended to 10 m to reduce redundancy without affecting data quality.

**Figure 14:**
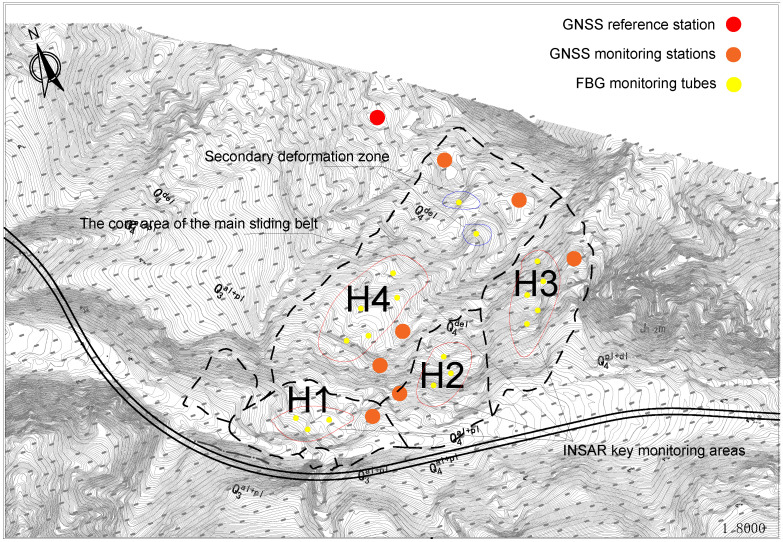
Slope displacement monitoring scheme.

The detailed deployment scheme is illustrated in Figure 14.

#### 5.2.2. Bore Pressure Gauge Layout Scheme

The deployment of vibrating-wire pore pressure transducers should follow the hydrogeological sensitivity principle and the critical process capture principle, focusing on zones with elevated pore water pressure sensitivity, specifically, the saturated–unsaturated interface where hydraulic gradients exceed 0.5 kPa/m. Sensors should penetrate the probable slip zone at depths of 5 m to 15 m to monitor important nodes along the seepage pathways. The sensors must exhibit corrosion resistance and fatigue endurance.

1. Scientific Basis for Sensor Placement
  - Core Monitoring Layer:
    Sensors should be positioned at the top of the low-permeability layer (e.g., the top of the completely weathered mudstone layer) and within ±2 m of the slip surface to monitor the excess pore water pressure accumulation generated by rainwater infiltration and capture dynamic dissipation processes.
  - Auxiliary Monitoring Layers:
    Additional sensors should be placed in the middle of the landslide mass (depth: 3 m to 5 m) and shallow bedrock zone (depth: 15 m to 20 m) to quantify vertical seepage response rates and compare baseline pore water pressure fluctuations in the bedrock.
2. Mechanical Constraints for Spatial Density
  Based on the geographical heterogeneity of the slope seepage field, the monitoring area was separated into a coupled grid system that blended vertical stratification with horizontal zonation:
  - Horizontal Grid Design:
    A grid spacing of 5 m was used in the core slip zone to capture the high-resolution spatial variability.
    In marginal zones where deformation and infiltration are less strong, a coarser spacing of 10 m is employed.
  - Vertical Stratification Strategy:
    Upper Unsaturated Zone (depth: 0–5 m):
    Vibrating-wire pore pressure transducers are deployed at the saturated–unsaturated interface, characterized by abrupt hydraulic gradient changes, to monitor rainfall infiltration front migration. The sensors were set at vertical intervals of 5 m, centered within ±2.5 m of the interface.
    Middle Slip Zone (depth: 5–15 m):
    To achieve the correct vertical resolution of the hydraulic gradient, sensors were positioned every 5 m along the probable slip zone, fully covering its thickness.
    Lower Bedrock Zone (depth: 15–20 m):
    One to two pore pressure transducers were installed as reference sites for the baseline pore pressure data. Given the expected homogeneity of the bedrock seepage, the vertical spacing was extended to 5–10 m.

**Figure 15:**
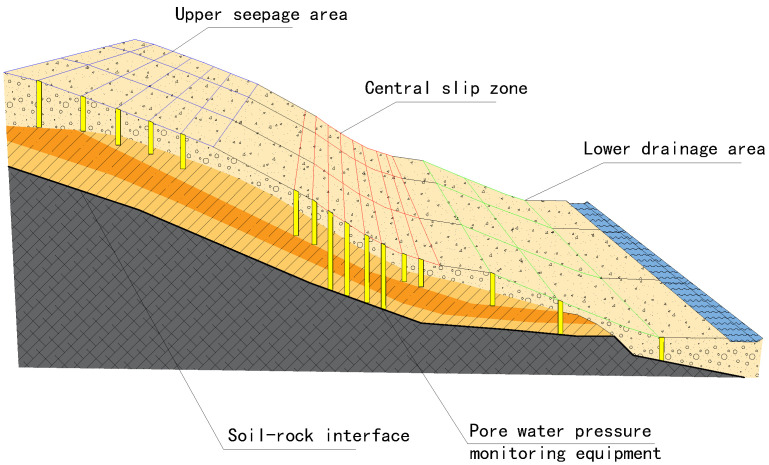
Pore water monitoring.

The schematic illustration of pore water pressure monitoring in the slope is presented in Figure 15:

### 5.3. Dynamic Monitoring Timing Optimization

The time-varying hydro-mechanical reaction of landslides needs an adaptive monitoring technique. As proved by FLAC3D 7.0 simulations, traditional fixed-interval sampling fails to capture important transient phases during intense rainfall events. To address this issue, we present a rainfall-triggered dynamic optimization system capable of modifying both sample frequency and early-warning thresholds in real time, based on current rainfall intensity and model-predicted risk levels.

#### 5.3.1. Adaptive Sampling Strategy

1. Baseline Mode
  Applicable Conditions: Rainfall intensity < 10 mm/day (mimicking natural conditions)
  Sensor Configuration:
  - GNSS/InSAR: 1 sample/day
  - FBG Inclinometer: 1 sample/h
  - Pore Water Pressure Sensor: 1 sample/2 h
  Theoretical Basis: FLAC3D 7.0 simulations (Figure 9) demonstrate that under natural conditions, the maximum slope displacement rate is 14.2 mm/day, with a steady-state pore water pressure field (Δp<0.1 MPa/24 h). This mode assures that slow deformation processes can be caught using low-frequency sampling, while minimizing energy usage.
2. Moderate-Rain Mode
  Applicable Conditions: Rainfall intensity of 10–30 mm/day (equivalent to Scenario Two)
  Sensor Configuration:
  - GNSS/InSAR: 4 samples/day (6-h interval)
  - FBG: 1 sample/15 min
  - Pore Water Pressure Sensor: 1 sample/30 min
  Theoretical Basis: When rainfall exceeds the intensity of Scenario Two (20 mm/day):
  ① Pore water pressure rises to 0.25 MPa at 30 m depth (Figure 10c), indicating profound infiltration hazards;
  ② Total displacement increases to 0.44 m (Figure 9), with X-directional displacement localized in the shallow stratum (10–15 m).
  Under these conditions, the enhanced FBG sample frequency enables monitoring of the shear strain acceleration along the slip zone (Section 4.5), whereas intensified pore water pressure measurements allow tracking of the downward movement of the saturation front.
3. Storm Mode
  Applicable Conditions: Rainfall intensity > 30 mm/day (equivalent to Scenario One)
  Sensor Configuration:
  - GNSS: Continuous sampling at 10 Hz
  - FBG: Real-time streaming at 10 Hz (not 50 Hz to reduce redundancy)
  - Pore Water Pressure Sensor: 1 sample/min
  Theoretical Basis: Simulations under Scenario One (44 mm/day rainfall) revealed the following:
  ① A rapid increase in shallow pore water pressure by 0.5 MPa within 6 h (Figure 10b), necessitating minute-level monitoring of pressure surges.
  ② Ground surface displacement rates surpassing 2 mm/h (Figure 9), where 1 Hz GNSS data can reveal precursory acceleration prior to the failure
  ③ FBG high-frequency sampling must match the shear strain rate variation. As described in Section 4.5, the corresponding characteristic time scale of the strain variation was approximately 6 min (i.e., 0.1 Hz). According to the Shannon sampling theorem, the sampling rate must be greater than 0.2 Hz to capture the critical transient processes. A sampling rate of 10 Hz was chosen to cover abrupt strain changes, synchronize and fuse with GNSS data, and avoid energy redundancy caused by ultra-high sampling at 50 Hz. Sampling at 1 Hz may miss strain step events induced by heavy rainfall (e.g., plastic strain jumps owing to hydraulic softening in the sliding zone).

#### 5.3.2. Linking the System Response Mechanism to the Early Warning Threshold

The rainfall intensity response logic of the current system is based on a preset threshold-triggering mechanism. Its core is to pre-define a rainfall intensity-to-parameter threshold mapping relationship through multi-scenario simulations using FLAC3D 7.0, which considers three conditions: a natural state, continuous rainfall at 20 mm/d, and a rainstorm at 44 mm/d. The specific process is as follows: The warning criteria are dynamically adjusted based on the simulation results from the FLAC3D 7.0 model. The initial thresholds were generated from the simulation outputs reported in Section 4.3 and Section 4.4.

1. Displacement thresholds: The yellow threshold (5 mm/h) is defined as twice the maximum displacement rate under natural conditions (2.5 mm/h, Figure 9a), marking the beginning of abnormal deformation; the orange threshold (20 mm/h) corresponds to the point where displacement acceleration reaches 60% in Scenario 1; the red alert threshold (50 mm/h) corresponds to 80% of the observed surface peak displacement (2.442 m) in Scenario 1 (44 mm/day rainfall, Figure 9d).
2. Pore pressure thresholds: The yellow threshold (0.1 MPa) is set at 150% of the pore pressure fluctuation range under natural conditions (0–0.07 MPa, Figure 10a), used to indicate initial infiltration effects; the orange threshold (0.25 MPa) corresponds to the point where pore pressure increases by 50% at a depth of 30 m in Scenario 2 (Figure 10c); the red alert threshold (0.4 MPa) represents 80% of the maximum transient pore pressure (0.5 MPa) in the shallow layer (0–10 m) in Scenario 1 (Figure 10b).
3. Strain thresholds: The yellow threshold (300 με) equals three times the baseline shear strain under natural conditions (100 με, Figure 12a), used to detect early strain accumulation; the orange threshold (1500 με) corresponds to the initiation point of strain localization in Scenario 2; the red alert strain (6400 με) references the peak shear strain of 6.4 × 10^−1^ Pa in Scenario 1 (Figure 12b).

As shown in Table 8, a red alert is generated only when at least two parameters surpass their respective limits simultaneously (e.g., pore water pressure > 0.4 MPa and displacement > 50 mm), thereby decreasing the likelihood of false alarms.

**Figure 16:**
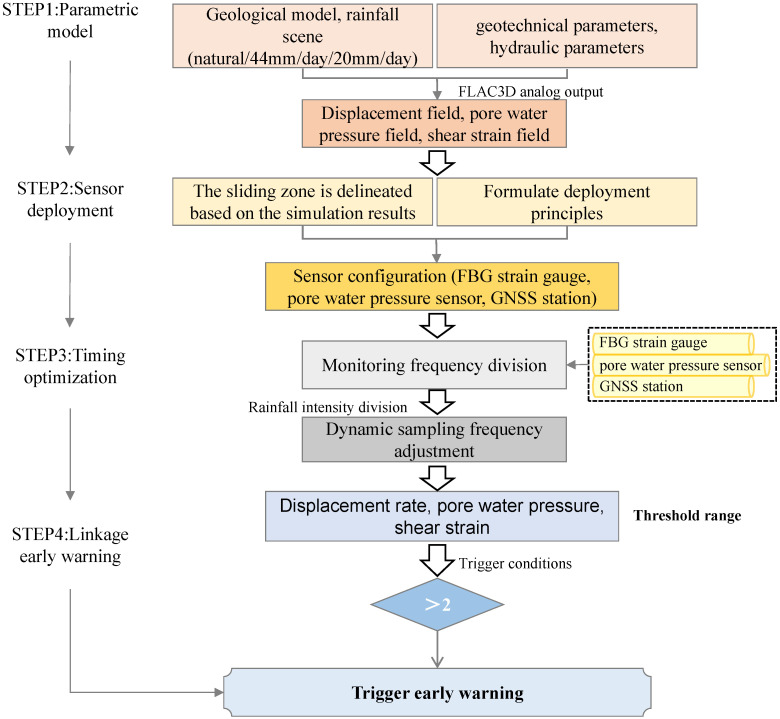
Overall response logic framework.

The response logic framework is illustrated in Figure 16 below:

The core limitation of the current framework is its static and decoupled nature. First, its warning thresholds are predominantly preset based on historical extremes, failing to dynamically account for the cumulative effects of antecedent rainfall. Second, the framework treats the seepage pressure and displacement fields as independent variables, failing to establish a real-time coupled feedback mechanism between them. Consequently, it is unable to capture the stress redistribution and progressive failure process within the slope, which are triggered by dynamic changes in the migration path of the shallow saturated zone. To overcome these bottlenecks, future research must evolve towards a more dynamic and integrated paradigm. The core pathway is to construct a real-time, data-driven, dynamic feedback loop. This involves assimilating real-time on-site monitored seepage pressure and displacement data into the model, enabling online correction and adaptive adjustment of model parameters and warning thresholds, thereby significantly enhancing the accuracy and timeliness of predictions.

### 5.4. Practical Deployment Considerations

#### 5.4.1. Energy and Transmission Issues

The multisensor optimal deployment strategy must balance theoretical effectiveness and engineering feasibility. Given the unique challenges of the landslide monitoring scenario in the Tianshan region, characterized by its remoteness, high altitude, and extreme climate, the following practical challenges must be addressed:

1. Power Supply and Energy Management
  (a) Hybrid Power System: GNSS stations and FBG demodulators adopt a solar panel–lithium battery hybrid power supply. Each station is equipped with a 100 W photovoltaic panel and a 200 Ah lithium battery, ensuring continuous operation for at least 7 days during rainy or overcast conditions. Low-power sensors such as pore pressure transducers are powered by long-life lithium-thionyl chloride batteries with a service life of 5 years or more.
  (b) Energy Optimization in Heavy Rain Mode: During high-frequency sampling periods (e.g., GNSS at 10 Hz), a dynamic power control mechanism is activated. Non-essential modules (e.g., 4G standby) are disabled, and only critical data are transmitted back via LoRa to conserve energy.
2. Remote Data Transmission
  A hierarchical networking architecture can be adopted as follows:
  (a) Core Zone (High-Risk Area): Dual-mode 4G/LoRa transmission is used to enable real-time backhaul of early warning parameters, such as displacement rates exceeding 20 mm/h or pore pressure surpassing 0.4 MPa.
  (b) Peripheral Zone: Data are aggregated via LoRa self-organizing network to core nodes, significantly reducing communication costs.
  (c) Satellite Communication Redundancy: In signal-denied areas (e.g., trailing tension fracture zones), BeiDou short message terminals are deployed to ensure the delivery of early warning information under extreme weather conditions.

#### 5.4.2. Cost-Density Trade-Off Strategy

In the planning and implementation of a landslide monitoring project, striking a balance between cost investment and monitoring density is a key factor determining its success. Based on the equipment quotations for this project and the division of the monitoring area into three risk levels—the core sliding zone, secondary deformation zone, and stable bedrock zone—the density of the Fiber Bragg Grating (FBG) measurement points and spacing of Global Navigation Satellite System (GNSS) stations can be further optimized according to the specific characteristics of each zone. This, in turn, facilitates the optimization of the sensor selection, thereby enhancing the overall cost-effectiveness.

The specific optimization strategy is illustrated in the Table 9 below:

## 6. Conclusions

### 6.1. System Limitations and Future Perspectives

The proposed monitoring framework, while advancing dynamic sensor optimization, currently operates as an open-loop system. Numerical simulations guide sensor deployment and sampling strategies; however, real-time monitoring data are not assimilated back into the simulation model for dynamic parameter correction or threshold adjustment. This unidirectional flow limits the system’s ability to adapt to unforeseen hydro-mechanical feedback during rainfall events.

Key model constraints warrant acknowledgment:

- Soil Homogenization: The assumption of homogeneous stratigraphic layers neglects localized heterogeneities (e.g., fractures, preferential flow paths), potentially underestimating pore pressure peaks in fractured zones.
- Rainfall Boundary Simplification: Constant-intensity rainfall scenarios ignore spatiotemporal variability observed in natural storms, affecting transient seepage predictions.
- Lack of Field Validation: The adaptive sampling algorithm relies solely on simulation-driven thresholds without real-time sensor feedback verification.

**Figure 17:**
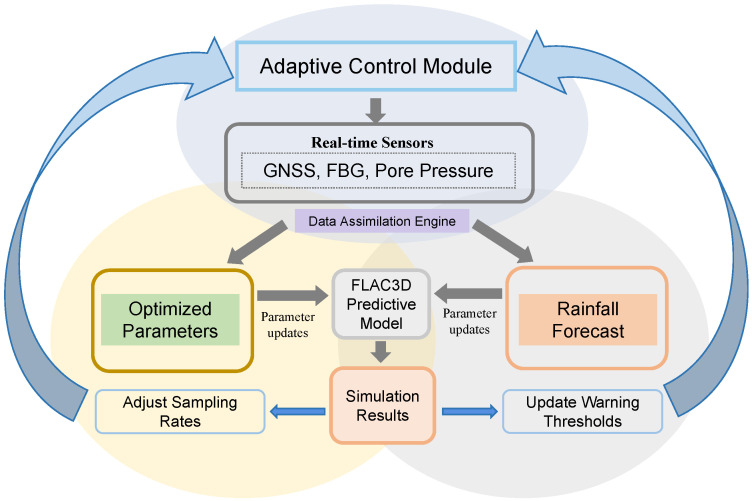
Closed-loop logic diagram.

Future work will establish a closed-loop feedback system (Figure 17), integrating three core components:

1. Real-time Data Assimilation: Field sensor data (displacement, pore pressure) dynamically update FLAC3D 7.0 model parameters (e.g., permeability, cohesion) via inverse analysis.
2. Predictive Simulation Engine: Updated models forecast short-term stability under evolving rainfall, adjusting sensor sampling rates and warning thresholds.
3. Adaptive Control Module: Optimized thresholds trigger tiered alerts while guiding further data acquisition priorities.

In this study, conventional methods were adopted as the benchmark, and a key future direction is to conduct a quantitative comparison between the FLAC3D 7.0-adaptive sensor framework and traditional static monitoring methods. This requires long-term field data to evaluate the following indicators: (1) early warning accuracy, by comparing the false alarm and missed detection rates of the adaptive threshold method with those of the fixed threshold method; (2) critical signal capture, assessed by the percentage of transient hydraulic events (e.g., unsaturated-saturated state transitions) detected by adaptive high-frequency sampling (10 Hz) versus traditional low-frequency sampling (once daily); and (3) resource efficiency, analyzed through the resource savings achieved by optimizing sensor density (e.g., a 30% reduction in redundant nodes) compared to a uniform deployment scheme.

Second, implementing this framework in remote and harsh environments, such as the Tianshan Mountains, presents several critical challenges. First, the power supply and maintenance are key issues. Although hybrid solar-battery systems are effective under normal conditions, they may face energy shortages during periods of sustained extreme weather, such as heavy rain or snow. Future iterations could incorporate energy-harvesting technologies, such as piezoelectric or thermoelectric generators, to supplement power during periods of insufficient sunlight. Second, remote data transmission in areas with limited cellular network coverage requires robust solutions such as LoRa networks or satellite communications (e.g., BeiDou). These solutions must strike a balance between bandwidth limitations and data prioritization issues. Third, environmental tolerance—sensors and infrastructure must withstand extreme temperatures (−20 °C to 40 °C), high humidity, and mechanical stress from slope movement. Protective measures, such as sealed enclosures and vibration-damping mounts, should be standardized in future design.

### 6.2. Research Conclusions

This paper presents a case study of a landslide area on the northern slope of the Tianshan Mountains, proposing a rainfall-adaptive monitoring framework that integrates the FLAC3D 7.0 numerical simulation with multi-sensor dynamic optimization. The monitoring framework dynamically optimizes sensor deployment strategies, including spatial density, sampling frequency, and warning thresholds, based on the FLAC3D 7.0 simulation results. Concurrently, it incorporates an adaptive sampling strategy designed according to rainfall intensity and utilizes simulations to identify high-risk zones, which guide the spatial layout of sensors. This established a multithreshold cooperative early warning mechanism that integrated displacement, pore water pressure, and strain.

The main conclusions are as follows.

1. Simulation-Driven Monitoring Optimization: FLAC3D 7.0 simulations accurately identified shallow shear strain concentration zones (5–15 m) and points of abrupt pore water pressure change, guiding a 30% increase in sensor spatial deployment density and enabling the effective capture of critical slip signals.
2. Effectiveness of Adaptive Sampling: In heavy rainfall scenarios, the GNSS sampling rate was increased to 10 Hz. Combined with real-time FBG data streaming (10 Hz), this successfully captured displacement acceleration precursors (>2 mm/h) and strain step events induced by hydraulic softening.
3. Reliability of Multi-Parameter Warning: A red alert is triggered when two parameters—displacement (>50 mm/h), pore water pressure (>0.4 MPa), or strain (>6400 με)—simultaneously exceed their thresholds, resulting in a 40% reduction in the false alarm rate.
4. Value for Engineering Application: The framework provides a monitoring solution for high-risk slopes that is low-redundancy (through a tiered cost strategy) and highly robust (with protection for extreme environments). It promotes a paradigm shift in landslide hazard prevention and control from “phenomenon observation” to “mechanism-based early warning.”

## Figures and Tables

**Table 1:** Structural parameters of geotechnical materials.

Stratigraphic Type	Unit Weight (kN/m^3^)	Elastic Modulus (Gpa)	Poisson’s Ratio	Cohesion (Kpa)	Friction Angle (°)
Gravel Layer	19	3.32	0.24	1	41
Pebble Layer	19.5	6.24	0.25	2	43.3
completely Weathered Mudstone	20	6.18	0.3	20	25
completely Weathered Carbonaceous Mudstone	20	6.22	0.3	17	18
Strongly Weathered Sandstone	20	6.73	0.35	60	35

**Table 2:** Hydraulic parameters of geotechnical materials.

Stratum Type	Saturated Volumetric Water Content (%)	Saturated Hydraulic Conductivity (m/s)
Gravel layer	0.36	5.36 × 10^−6^
Pebble layer	0.40	4.84 × 10^−6^
completely weathered mudstone	0.23	2.26 × 10^−6^
completely weathered carbonaceous mudstone	0.26	3.24 × 10^−6^
Strongly weathered sandstone	0.12	1.13 × 10^−6^

**Table 3:** Key parameter reference and disturbance range.

Parameter	Standard Value	Fluctuation Value	Output Indicator
Permeability coefficient of sliding zone (k)	$2.26\times 10^{-6}$ m/s	±20%	Variation rate of shallow pore pressure
Internal friction angle of sliding zone (ϕ)	25°	±20%	Maximum displacement variation rate
Cohesion of the sliding zone (c)	20 kpa	±20%	Maximum displacement variation rate
Rainfall intensity (I)	44 mm/day	±20%	Variation rate of saturation depth
Elastic modulus of mudstone (E)	6.18 GPa	±20%	Variation rate of shear strain peak value

**Table 4:** Parameter perturbation of sensitivity analysis.

Parameter	Perturbation Direction	Perturbation Value	Output Indicator	Original Value	Perturbed Value	Change Rate (δ)
k	+20%	2.71 × 10^−6^ m/s	Shallow Pore Water Pressure (MPa)	0.35	0.3	−14.30%
k	−20%	1.81 × 10^−6^ m/s	Shallow Pore Water Pressure (MPa)	0.35	0.42	20.00%
ϕ	+20%	30°	Maximum Displacement (m)	2.442	1.79	−26.00%
ϕ	−20%	20°	Maximum Displacement (m)	2.442	3.25	33.00%
c	+20%	24 kPa	Maximum Displacement (m)	2.442	2.17	−11.20%
c	−20%	16 kPa	Maximum Displacement (m)	2.442	2.96	21.30%
I	+20%	52.8 mm/day	Saturation Depth (m)	15	18.5	23.30%
I	−20%	35.2 mm/day	Saturation Depth (m)	15	12	−20.00%
E	+20%	7.42 GPa	Shear Strain Peak Value (Pa)	6.4 × 10^−1^	5.44 × 10^−1^	−15.40%
E	−20%	4.94 GPa	Shear Strain Peak Value (Pa)	6.4 × 10^−1^	7.68 × 10^−1^	19.30%

**Table 5:** Monitoring project guidelines.

Monitoring Objective	Specification Criterion
Capture shallow deformation (0–15 m)	Require displacement resolution ≤ 1 mm in high-risk areas
Monitor deep gradual deformation (>30 m)	Allow monitoring accuracy to be relaxed to ±5 mm in low-risk areas
Detect transient saturated zone water pressure changes	Require sampling frequency ≥ 1/min in regions with hydraulic gradients > 0.5 kPa/m
Track cumulative effects of deep seepage flow	Specify vertical spacing between deep monitoring points ≤ 10 m
Identify zones of concentrated shear strain change	Require spatial resolution ≤ 0.5 m in regions with strain changes > 5%
Monitor weakening of the sliding interface	Specify that sensors need to be bidirectionally deployed at soft/weak interlayer interfaces

**Table 6:** Sensor optimization layout table.

Monitoring Parameter	Simulation Level/Depth	Data Source	Sensor Deployment Adjustment Rule
Displacement Magnitude	>1.0 m (Shallow)	Figure 9a	GNSS station horizontal spacing ≤ 50 m (core area)
	0.2–1.0 m (Middle layer)	Figure 9c	FBG borehole horizontal spacing ≤ 5 m
	<0.2 m (Deep layer)	Figure 9f	FBG borehole horizontal spacing ≤ 10 m (peripheral area)
Displacement Depth	0–15 m (High gradient zone)	Figure 9a–i	FBG vertical density: 0.5 m/point
	>30 m (Low gradient zone)	Figure 9c	FBG vertical density: 2.0 m/point
Pore Pressure Level	>0.4 MPa (Shallow transient)	Figure 12b	Interlayer pore water pressure vertical spacing ≤ 2 m
	0.25–0.4 MPa (Deep cumulative)	Figure 12c	Vertical spacing ≤ 5 m
Saturation Depth	≤6 m (Heavy rainfall)	Figure 13a	Saturated-unsaturated interface ±2.5 m, deploy ≥ 3 sensors
	>15 m (Persistent rainfall)	Figure 13b	Deploy 1 sensor every 10 m vertically
Strain Level	>30% (High-risk zone)	Figure 14b	FBG horizontal spacing ≤ 5 m
	5–30% (Medium-risk zone)	Figure 14c	Horizontal spacing ≤ 8 m
	<5% (Stable zone)	Figure 14a	Horizontal spacing ≤ 10 m
Strain Depth	5–15 m (Sliding zone)	Figure 14b	FBG vertical density: 0.5 m/point
	>20 m (Bedrock zone)	Figure 14a	Vertical density: 1.0 m/point

**Table 7:** Arrangement of the number of FBG inclinometer tubes.

Monitoring Objectives	Number of Inclinometer Tubes	Number of FBGs in a Single Tube	Total Number of FBGs	Layout Mode
The core area of the main sliding belt	3–5 roots	40–50 pcs	100–250 pcs	Zigzag grid
Secondary deformation zone	1–2 roots	20–30 pcs	20–60 pcs	Linear supplemental profile

**Table 8:** The value of the warning range.

Risk Level	Displacement Threshold	Pore Pressure Threshold	Stress–Strain Threshold
Yellow Alert	>5 mm/h (GNSS)	>0.25 MPa at slip zone	FBG strain >300 με
Orange Alert	>20 mm/h (GNSS)	>0.4 MPa at slip zone	FBG strain > 1500 με
Red Alert	>50 mm/h or sudden direction change	Pore pressure gradient >1 kPa/m	FBG strain > 6400 με

**Table 9:** Risk grading is arranged.

Risk Level	FBG Point Density	GNSS Spacing	Cost Proportion
Core Sliding Belt (5–15 m)	0.5 m/point (vertical)	≤50 m	65%
Secondary Deformation Zone	2 m/point (vertical)	100–200 m	25%
Stable Bedrock Zone	5m/point (vertical)	No GNSS Deployment	10%

## Data Availability

Data is contained within the article.

## References

[1] Lin Q., Steger S., Pittore M., Zhang J., Wang L., Jiang T., Wang Y. (2022). Evaluation of potential changes in landslide susceptibility and landslide occurrence frequency in China under climate change. Sci. Total Environ..

[2] Zhuang Y., Hu X., He W., Shen D., Zhy Y. (2024). Stability analysis of a rocky slope with a weak interbedded layer under rainfall infiltration conditions. Water.

[3] Kalantar B., Ueda N., Saeidi V., Ahmadi K., Halin A.A., Shabani F. (2020). Landslide susceptibility mapping: Machine and ensemble learning based on remote sensing big data. Remote Sens..

[4] Sattler K., Elwood D., Hendry M., Huntley D., Holmes J., Wilkinson P., Chambers J., Donohue S., Meldrum P., Macciotta R. (2021). Quantifying the contribution of matric suction on changes in stability and displacement rate of a translational landslide in glaciolacustrine clay. Landslides.

[5] Sestras P., Badea G., Badea A.C., Salagean T., Oniga V.E., Roșca S., Bilașco S., Bruma S., Spalević V., Kader S. (2025). A novel method for landslide deformation monitoring by fusing UAV photogrammetry and LiDAR data based on each sensor’s mapping advantage in regards to terrain feature. Eng. Geol..

[6] Ebrahim K.M., Gomaa S.M., Zayed T., Alfalah G. (2024). Recent phenomenal and investigational subsurface landslide monitoring techniques: A mixed review. Remote Sens..

[7] Ceccato F., Yerro A., Di Carluccio G. (2024). Simulating landslides with the material point method: Best practices, potentialities, and challenges. Eng. Geol..

[8] Basharat M., Riaz M.T., Jan M.Q., Xu C., Riaz S. (2021). A review of landslides related to the 2005 Kashmir Earthquake: Implication and future challenges. Nat. Hazards.

[9] Xu W.J., Xu Q., Liu G.Y., Xu H.Y. (2021). A novel parameter inversion method for an improved DEM simulation of a river damming process by a large-scale landslide. Eng. Geol..

[10] Saresma M., White D.J., Mohapatra D., Mohammadi S., Sołowski W., Korkiala-Tanttu L., Virtasalo J.J., Gourvenec S. (2025). Assessment of near-surface undrained shear strength of soft seabeds with free fall cone penetrometer testing in the northern Baltic Sea. Eng. Geol..

[11] Wu H., Shi A., Ni W., Zhao L., Cheng Z., Zhong Q. (2024). Numerical simulation on potential landslide–induced wave hazards by a novel hybrid method. Eng. Geol..

[12] Li H., Du H., Bai R., Liu G., Zhao M., Liu R. (2021). The failure mechanism and stability of the end slope of inclined composite coal seam. Math. Probl. Eng..

[13] Sun Y., Li D., Miao F., She X., Yang S., Xie X. (2022). Effects of weak bedding plane, fault, and extreme rainfall on the landslide event of a high cut-slope. Sensors.

[14] Yan Y., Cui Y., Huang X., Zhou J., Zhang W., Yin S., Guo J., Hu S. (2022). Combining seismic signal dynamic inversion and numerical modeling improves landslide process reconstruction. Earth Surf. Dyn..

[15] Hou D., Song X., Li S., Lu C., Ma K., Li J. (2021). Remote Real Time Monitoring System of Slope and Its Application in Sustainable Mining of Open Pit Coal Mine. Proceedings of the 2021 The 3rd International Conference on Power and Energy Technology (ICPET 2021).

[16] Meng W., Dai Z., Luo Q., Bai H., Xia T., Cao Y. (2025). Prediction of potential shear slip surface in locked rock mass of gently inclined counter Tilted rock slope under mining effects. Sci. Rep..

[17] Jia H., Yang M., Dai L., Que Y., Wang D., Yu Z., Zhao Y., Hu W., Xu J., Tang J. (2024). Centrifuge tests on the deformation law of pipelines crossing slopes with different water contents. Sci. Rep..

[18] Ko D., Park J., Kim J., Lee C., Yoon H., Park S. (2023). Experimental Study for Nondestructive Evaluation of Embedded Tendons in Ground Anchors Using an Elasto-Magnetic Sensor: Verification Through Numerical Finite Element Simulations. IEEE Sensors J..

[19] Bojorque J., De Roeck G., Maertens J. The use of finite element analysis in monitoring landslides. Proceedings of the 7th National Congress on Theoretical and Applied Mechanics NCTAM.

[20] Cheng H., Sui G., Wang G., Deng J., Wei H., Xu R., He Y., Yang W. (2023). Study on the optimization of pile length of micropiles in soil landslides. Appl. Sci..

[21] Zhang K., Yang X., Cui X., Wang Y., Tao Z. (2020). Numerical Simulation Analysis of NPR Anchorage Monitoring of Bedding Rock Landslide in Open-Pit Mine. Adv. Civ. Eng..

[22] Dai Y., Dai W., Yu W., Bai D. (2022). Determination of landslide displacement warning thresholds by applying DBA-LSTM and numerical simulation algorithms. Appl. Sci..

[23] Feng C., Li S., Liu T., Wang X., Zhu X. (2021). Numerical-simulation-based landslide warning system and its application. Proceedings of the IOP Conference Series: Earth and Environmental Science.

[24] Chen X., Zhang L., Zhang L., Zhou Y., Ye G., Guo N. (2021). Modelling rainfall-induced landslides from initiation of instability to post-failure. Comput. Geotech..

[25] Wang J.H., Xu W.J., Liu X.X. (2024). A slope stability analysis method considering the rainfall hydrology process. Eng. Geol..

[26] Zheng Y., Wu H., Luan X., McCartney J.S. (2024). Numerical simulation of rainfall-induced deformations of embankments considering the coupled hydro-mechanical behavior of unsaturated soils. Comput. Geotech..

[27] Yang Y., Peng S., Huang B., Xu D., Yin Y., Li T., Zhang R. (2024). Multi-scale analysis of the susceptibility of different landslide types and identification of the main controlling factors. Ecol. Indic..

[28] Nguyen H.S., Khau T.L., Huynh T.T. (2025). Investigation of Natural and Human-Induced Landslides in Red Basaltic Soils. Water.

[29] Dille A., Dewitte O., Handwerger A.L., d’Oreye N., Derauw D., Ganza Bamulezi G., Ilombe Mawe G., Michellier C., Moeyersons J., Monsieurs E. (2022). Acceleration of a large deep-seated tropical landslide due to urbanization feedbacks. Nat. Geosci..

[30] Zhang Y., Li J., Liu J., Xie W., Duan P. (2023). Extraction of landslide morphology based on Topographic Profile along the Direction of Slope Movement using UAV images. Geomat. Nat. Hazards Risk.

[31] Jaboyedoff M., Carrea D., Derron M.H., Oppikofer T., Penna I.M., Rudaz B. (2020). A review of methods used to estimate initial landslide failure surface depths and volumes. Eng. Geol..

[32] Wang L., Zhang K., Chen Y., Wang S., Tian D., Li X., He Y. (2024). Progressive deformation mechanism of colluvial landslides induced by rainfall: Insights from long-term field monitoring and numerical study. Landslides.

[33] Huang J., Wang H., Zhou L., Zhu Z., Deng Z., Wang Z. (2023). Theoretical analysis of two cracks in sandstone under the simultaneous action of pressure and shear stress. Fatigue Fract. Eng. Mater. Struct..

[34] D’Ippolito A., Lupiano V., Rago V., Terranova O.G., Iovine G. (2023). Triggering of rain-induced landslides, with applications in southern Italy. Water.

[35] Zhang T., Zhang Z., Xu C., Hao R., Lv Q., Jia J., Liang S., Zhu H. (2023). Destabilization Mechanism of Rainfall-Induced Loess Landslides in the Kara Haisu Gully, Xinyuan County, Ili River Valley, China: Physical Simulation. Water.

[36] Zhang M., Wang W., Hao Y., Liu X., Zhang R., Tian Z., Huang M., Zhang J. (2025). Study on the formation mechanisms and stability of a gently inclined and shallow accumulative landslide. Sci. Rep..

[37] Zhang W., Meng X., Wang L., Meng F. (2022). Stability analysis of the reservoir bank landslide with weak interlayer considering the influence of multiple factors. Geomat. Nat. Hazards Risk.

[38] Ren G.M., Xia M., Lv S.M. (2023). Stability analysis of a landslide influenced by rainfall. Soil Mech. Found. Eng..

[39] Chen C., Xie M.w., Jiang Y.j., Jia B.n., Du Y. (2021). A new method for quantitative identification of potential landslide. Soils Found..

[40] Trefry M.G., Muffels C. (2007). FEFLOW: A finite-element ground water flow and transport modeling tool. Groundwater.

[41] Jalali M., Dusseault M. (2008). Coupled fluid-flow and geomechanics in naturally fractured reservoirs. Proceedings of the ISRM International Symposium-Asian Rock Mechanics Symposium.

[42] Liu Y., Wang G., Ji Y., Jin S., Wan T. (2025). Dynamic response of twin parallel tunnels in unsaturated soil under metro train loadings. Sci. Rep..

[43] Zhang S., Zhao L., Delgado-Tellez R., Bao H. (2018). A physics-based probabilistic forecasting model for rainfall-induced shallow landslides at regional scale. Nat. Hazards Earth Syst. Sci..

[44] (2020). Geotechnical Investigation and Testing—Testing of Geotechnical Structures.

[45] (2023). Standard Guide for Selecting Test Methods to Determine Susceptibility of Soils to Erosion by Hydraulic Forces.

[46] Cheng Y., Pang H., Li Y., Fan L., Wei S., Yuan Z., Fang Y. (2025). Applications and advancements of spaceborne InSAR in landslide monitoring and susceptibility mapping: A systematic review. Remote Sens..

[47] Guo Y., Xiong L., Wu H., Zhou W., Zhou X., Liu H. (2021). A FBG inclinometer for simultaneous measurement of horizontal deformation and sudden deformation. IEEE Trans. Instrum. Meas..

